# Users' and health service providers' perception on quality of laboratory malaria diagnosis in Tanzania

**DOI:** 10.1186/1475-2875-10-78

**Published:** 2011-04-06

**Authors:** Yahya A Derua, Deus RS Ishengoma, Rwehumbiza T Rwegoshora, Filemoni Tenu, Julius J Massaga, Leonard EG Mboera, Stephen M Magesa

**Affiliations:** 1 National Institute for Medical Research, Amani Centre, P. O. Box 81, Muheza, Tanzania; 2 National Institute for Medical Research, Tanga Centre, P. O. Box 5004, Tanga, Tanzania; 3 National Institute for Medical Research, Headquarters, P. O. Box 9653, Dar es Salaam, Tanzania

## Abstract

**Background:**

Correct diagnosis of malaria is crucial for proper treatment of patients and surveillance of the disease. However, laboratory diagnosis of malaria in Tanzania is constrained by inadequate infrastructure, consumables and insufficient skilled personnel. Furthermore, the perceptions and attitude of health service providers (laboratory personnel and clinicians) and users (patients/care-takers) on the quality of laboratory services also present a significant challenge in the utilization of the available services. This study was conducted to assess perceptions of users and health-care providers on the quality and utilization of laboratory malaria diagnostic services in six districts from three regions in Tanzania.

**Methods:**

Questionnaires were used to collect information from laboratory personnel, clinicians and patients or care-takers.

**Results:**

A total of 63 laboratory personnel, 61 clinicians and 753 patients/care-takers were interviewed. Forty-six (73%) laboratory personnel claimed to be overworked, poorly motivated and that their laboratories were under-equipped. About 19% (N = 12) of the laboratory personnel were lacking professional qualification. Thirty-seven clinicians (60.7%) always requested for blood smear examination to confirm malaria. Only twenty five (41.0%) clinicians considered malaria microscopy results from their respective laboratories to be reliable. Forty-five (73.8%) clinicians reported to have been satisfied with malaria diagnostic services provided by their respective laboratories. Majority (90.2%, N = 679) of the patients or care-takers were satisfied with the laboratory services.

**Conclusion:**

The findings show that laboratory personnel were not satisfied with the prevailing working conditions, which were reported to undermine laboratory performance. It was evident that there was no standard criteria for ordering malaria laboratory tests and test results were under-utilized. Majority of the clinicians and patients or care-takers were comfortable with the overall performance of laboratories, but laboratory results were having less impact on patient management.

## Background

Laboratory diagnosis is an important component of case management and control of malaria [1]. However, inaccurate microscopy and symptomatic diagnosis of malaria occur frequently in most endemic countries including Tanzania [2-4]. This has been attributed to inadequate financial resources to support diagnostic services, insufficient skilled laboratory personnel and low reproducibility of laboratory results [5-7]. In these countries, clinical guidelines have been developed and recommended for symptomatic and differential diagnosis of malaria. Some of these guidelines have been reported to be highly sensitive in detecting malaria cases but their specificity is low because malaria symptoms are quite often similar to those of other febrile tropical diseases [8].

Malaria diagnosis based on clinical signs and low accuracy of malaria microscopy have resulted in over-diagnosis of malaria which carries a risk of unnecessary use of anti-malarial drugs (which is the main cause of parasite resistance) and masking other underlying causes of febrile illnesses [4]. Likewise under-diagnosis of malaria may result in delayed treatment and progression into severe cases with fatal consequences. Using the current clinical guidelines, at low prevalence of malaria symptomatic diagnosis carries a low risk of missing malaria cases but with substantial over-prescription of anti-malarial drugs [9]. Although symptomatic diagnosis of malaria has been considered reasonable in resource poor settings with high malaria transmission where laboratory infrastructure is inadequate [10], the current level of misdiagnosis has been found to be unsustainable particularly after introduction of the more expensive artemisinin-based combination drugs [11,12].

In those areas with adequate malaria diagnostic services, perceptions and practices of clinicians stand to be important barriers to effective utilization of laboratory results. Chandler *et al *[13,14] have shown that malaria diagnostic practices have a strong link to clinical and contextual factors where malaria is strongly promoted as a disease that could be easily diagnosed clinically. It can be argued that, shortage of qualified laboratory personnel and inadequate quality assessment systems contribute significantly in eroding the confidence of clinicians in applying laboratory results [15]. However, with optimal malaria laboratory testing, it has been recognized that the test results may remain underutilized in managing febrile illnesses [16,17]. Studies assessing perception of patients or care-takers [18,19] and clinicians [20] with respect to malaria diagnosis have elaborated among other things two important scenarios; that patients prefer laboratory testing before anti-malarial drug prescription and sometimes malaria laboratory results may have little influence in prescription practices by clinicians. The reasons behind underutilization of malaria laboratory results by clinicians need to be investigated and addressed accordingly in order to build a culture of rational management of malaria.

In view of poor quality of laboratory services, the Tanzanian Ministry of Health and Social Welfare planned to implement a national improvement framework aiming at improving malaria diagnosis at different levels of health care delivery system. In support of the improvement programme, this study reports the findings of a survey conducted to assess the perceptions of laboratory users and health service providers on malaria diagnosis in six districts of Tanzania.

## Methods

### Study site and participants

This study was conducted between January and March 2007 in six districts of Tanzania namely; Muheza, Tanga City, Mpwapwa, Dodoma Rural, Iringa Rural, and Iringa Municipality covering a total of 36 health facilities. Tanga and Muheza districts are located in north-eastern part of Tanzania while Dodoma Rural and Mpwapwa districts are in the central part of the country. Iringa Rural and Iringa Municipality are located in the southern highlands of Tanzania. Details of study site and sampling of the health facilities is presented elsewhere [6]. Briefly, in each district, a total of six health facilities with functional laboratories (defined as a laboratory with equipment and personnel capable of performing malaria diagnosis by microscopy at the time of the investigators' visits) were selected for the survey. Study participants included laboratory personnel, clinicians and patients or care-takers attending the respective health facilities on the days of investigators' visit.

### Data collection procedures

Information on the number and qualifications of clinicians and laboratory personnel working in the study health facilities were collected using questionnaires from the head of the facility and laboratory, respectively. Questionnaires were also used to assess views and perceptions of laboratory personnel and clinicians on malaria diagnostic services provided in their respective laboratories. Laboratory personnel were also interviewed to obtain information on their training qualifications and their perceived constraints regarding laboratory malaria diagnosis. Other information collected included job satisfaction, refresher training courses attended and future training requirements for appropriate laboratory diagnosis of malaria. From clinicians, further information on their professional training, reasons for requesting laboratory malaria investigations, reliability of the test results and use of laboratory results in malaria case management was collected.

Patients or their care-takers (in case of children below 15 years) attending each of the study facilities were interviewed using a questionnaire to obtain demographic information, time spent to wait for laboratory results and their perceptions regarding the results as well as the quality of malaria diagnostic services provided. To get an estimation of waiting time, patients or their care-takers were questioned on how long they have been waiting for laboratory results from the time the test was taken to when results were made available. All questionnaires in this survey were administered face to face where the interviewer presented a series of standardized questions orally to the interviewee. To validate the responses provided by the respondents, questions with yes/no responses were followed by a cross-check request for further explanations. Information on the clinical diagnosis of each patient as written by attending clinician on the patient's card was recorded by the investigators at the entrance to the laboratory. Details of the tests performed and laboratory results were recorded from the patient's card when leaving the consultation room.

### Data analysis

The data obtained was managed using SPSS software version 11.5 (SPSS Inc., Chicago, IL, US) and later analysed using STATA version 8.0 (Stata Corp, College Station, TX). Qualitative data were summarized in themes or texts and presented as proportions of different variables or in tabular format. Different categorical variables were compared using Chi-square test and p-value ≤ 0.05 was considered significant.

## Results

### Laboratory personnel

A total of 63 laboratory personnel were interviewed whereby laboratory technicians, laboratory assistants and laboratory attendants accounted for 15.9% (N = 10), 55.6% (N = 35) and 28.6% (N = 18) of the personnel, respectively (Table 1). A mean of two laboratory personnel were interviewed per health facility (with a range of one to six personnel per facility). Twenty-one laboratories (58.3%, n = 36) had only one laboratory personnel. Of these particular laboratories, 12 (57.1%), eight (38.1%) and one (4.8%) were each run by a laboratory assistant, laboratory attendant and laboratory technician, respectively. Of all interviewed laboratory personnel, one (1.6%) had first degree and seven (11.1%) had diplomas, all obtained after three years post-secondary school training in medical laboratory technology. Forty-three laboratory personnel (68.3%) had certificates in medical laboratory technology, of which 33 and 10 were obtained after two and one year of training, respectively. Twelve (19.0%) laboratory personnel (four laboratory assistants and eight attendants) had not received any formal training.

**Table 1:** Number (%) of interviewed laboratory personnel from 36 health facilities in six districts of Tanzania

Cadre of Personnel	Muheza	Tanga	Dodoma Rural	Mpwapwa	Iringa Rural	Iringa Municipality	Total
Lab Technicians	1 (8.3)	5 (35.7)	1 (14.3)	0 (0)	2 (25.0)	1 (7.7)	10 (15.9)

Lab assistant	9 (75.0)	6 (42.9)	2 (28.6)	6 (66.7)	5 (62.5)	7 (53.9)	35 (55.6)

Lab attendant	2 (16.7)	3 (21.4)	4 (57.1)	3 (33.3)	1 (12.5)	5 (38.5)	18 (28.6)

Total	12 (100)	14 (100)	7 (100)	9 (100)	8 (100)	13 (100)	63 (100)

Lab = Laboratory

Only 36 (57.1%) laboratory personnel had attended at least one refresher course since their last graduation (years of service up to the date of visit ranged from 1 - 36, with a mean of 13.7 years). Of these, 16 (44.4%) had attended only one course, 13 (36.1%) attended two, six (16.7%) attended three and only one (2.8%) had attended four courses. With respect to course contents, 22 (61.1%, n = 36) laboratory personnel attended at least one malaria related courses and the rest (38.9%) attended one or more non-malaria courses.

Thirty-six (57.1%) laboratory personnel reported to have been satisfied with their job. Out of these, 23 (63.9%) said that they were satisfied because they liked the job, seven (19.4%) said that they had better working environment and two (5.6%) felt that they had better opportunities. Four laboratory personnel (11.1%) could not give reasons for their job satisfaction. For those who were not satisfied with their job, 18 (66.7%) said that they lacked training opportunities, had poor remuneration/motivation and they were overworked while seven (25.9%) reported poor working environment as a main reason for their dissatisfaction. Two (7.4%) laboratory personnel could not give any reason for not being satisfied with their job. The level of job satisfaction was not significantly different among laboratory personnel of different cadres (laboratory technicians, assistants and attendants; p = 0.168) and type of health facility they were based (hospitals, health centres or dispensaries; p = 0.53). However, there was a strong tendency towards satisfaction among laboratory staff who were geographically located in rural (72%, 18/25) as compared to those from urban areas (47.4%,18/38) (p = 0.053). This trend was supported by complaints from the majority (44.7%, 17/38) of laboratory personnel working in urban areas that they were overworked, poorly motivated, had few training opportunities and their laboratories were inadequately supplied with equipment and consumables. The laboratory personnel (64%, 16/25) in rural areas were relatively comfortable with the prevailing working conditions.

Forty-six (73.0%) laboratory personnel reported lack of equipment and/or reagents, lack of training or retraining, heavy workload and poor motivation as the major constraints to the performance of their laboratories. Six (9.5%) reported that laboratory personnel were under-qualified or incompetent while five (7.9%) mentioned shortage of working space or facilities, electricity and water as the main constraints. Six (9.5%) respondents did not report any constraints. Regarding future training requirements, 47 (81.0%) laboratory personnel indicated that newly and improved malaria diagnostic techniques, such as rapid diagnostic tests (RDT), quantitative buffy coat (QBC), acridine orange (AO) and Giemsa staining technique were needed. Two (3.4%) laboratory personnel suggested introduction of standardized parasite counting method. Non-malarial diagnostic tests were also recommended by laboratory personnel that included bacterial culturing (3.4%, N = 2), HIV testing (1.7%, N = 1) and Leishman stain technique (1.7%, N = 1). In addition, six (10.3%) laboratory personnel recommended that advanced laboratory training need to be introduced and five did not have any recommendations. To improve malaria diagnostic services, thirty seven (58.7%) laboratory personnel recommended for recruitment of more laboratory personnel, procurement of more laboratory equipment, training of available laboratory personnel and providing better remuneration. Ten (15.9%) laboratory personnel recommended frequent refresher courses and eight (12.7% were of the view that adoption of new diagnostic techniques will improve malaria diagnosis. Of the remaining eight laboratory personnel, six (9.5%) gave no recommendations, one (1.6%) recommended community sensitization to accept laboratory results and the last one (1.6%) advised on policy reviews by the Ministry of Health to recognize service offered by laboratory assistants and attendants.

### Clinicians

A total of 61 clinicians were interviewed. Fifty (82.0%) were clinical officers with diploma training in clinical medicine, two (3.3%) were assistant medical officers with advanced diploma in clinical medicine; one (1.6%) was a medical doctor and three (4.9%) were specialists with post-graduate training in medicine. Four nurses (6.6%, one auxiliary nurse without any formal training in nursing) and one assistant clinical officer (1.6%) with a certificate in medicine were also interviewed. Thirty-seven (60.7%) clinicians always requested blood smear examination to confirm malaria and 24 (39.3%) reported to request for laboratory malaria tests infrequently. For clinicians who always requested for blood smears examination (n = 37), gave three reasons for doing so, as to confirm diagnosis (64.9%, N = 24), for differential diagnosis (29.7%, N = 11) or upon patients' request for the test (5.4%, N = 2). For the clinicians who requested blood smear examination less frequently (n = 24), 15 (62.5%) mentioned that it depended on patient history or symptoms and three (12.5%) said that requests were made only when the laboratory was open. Three (12.5%) clinicians said that they did request blood smear examination to confirm diagnosis, two (8.3%) asked for blood smear examination when attending adult patients with fever while one (4.1%) requested for malaria diagnosis tests when managing fewer patients.

Twenty-five (41.0%) clinicians reported that the results from their laboratories were very reliable while 34 (55.7%) said that the results were just reliable and two (3.3%) said that the results were unreliable. Of those who reported that the results were very reliable, 13 (52.0%) said that their laboratories had competent and motivated laboratory personnel, one (4%) said that they had reliable equipment, one (4%) said that their laboratory uses more than one method for malaria diagnosis (i.e. blood smears and QBC) and the rest 10 (40.0%) gave no reasons. Among 34 clinicians who reported that results were just reliable, 16 (47.1%) said that parasites can be missed because of the complex nature of the parasite life cycle, eight (23.5%) mentioned that their laboratory staff were incompetent, four (11.8%) said that laboratory staff were overworked, three (8.8%) reported that it was because of poor state of microscopes and/or expired reagents and three (8.8%) gave no reasons. For the two clinicians who said that the results were unreliable, one said that parasites might be missed during smear examination and the other did not give any reason. Forty-five (73.8%) clinicians reported to have been satisfied with malaria diagnostic services provided by their laboratories. However, only 25 (41.4%) clinicians reported to always honour and use laboratory results for management of patients while 35 (57.4%) sometimes honoured the results and one (1.6%) clinician rarely honoured laboratory results.

### Patients or care-takers

A total of 753 patients or care-takers were interviewed. There were more female (68.4%, N = 515) than male (31.6%, N = 238) respondents. The majority of the respondents (66.5%, N = 501) had primary school education while 124 (16.5%) had secondary school education, 16 (2.1%) had post secondary school education and the remaining 112 (14.9%) had no formal education. Over half (53.3%) of the respondents were peasants while students, merchants, employees and those involved in other subsistence activities accounted for 14.3%, 13.1%, 8.5% and 10.8% of all respondents, respectively.

Slightly over half (56.6%) of the patients had fever as the main clinical symptoms and 94 (12.5%) were provisionally diagnosed (based on history and clinical examination) to have malaria prior to laboratory investigations (Table 2). Blood slide examination was requested by the attending clinicians and taken to confirm malaria from 716 (95.1%) patients. Thirty-seven patients (4.9%) requested for blood slide examination to the laboratory personnel without prior consultation with clinicians. Out of these, 17 were tested based on the decision of laboratory personnel while 20 patients were not tested to detect if they had malaria parasites. Of the 733 examined patients, 245 (33.4%) were reported to have malaria parasites. When fever symptom was related to malaria, the ability of fever to predict true positive cases of malaria (positive predictive value) using microscopy as a gold standard was found to be 33.8% with sensitivity and specificity of 56.7% and 43.1%, respectively. Of the 94 people who were diagnosed to have malaria based on clinical presentation, 86 had their blood examined for confirmation of malaria parasites and only 35 (40.7%) were detected to have malaria parasites by microscopy. With respect to blood slide results, 99.2% (243/245) of slide positive and 83.6% (408/488) of slide negative patients were satisfied with laboratory results.

**Table 2:** Clinical symptoms and provisional diagnosis as written by attending clinicians

Symptoms/signs/tentative diagnosis	Tanga (%), n = 290	Dodoma (%), n = 237	Iringa (%), n = 226	Total (%), n = 753
Fever	129 (44.5)	140(59.1)	157 (68.9)	426 (56.6)

Headache	65 (22.4)	51(21.5)	67 (29.4	183 (24.3)

Malaria	46 (15.9)	37(15.6)	11 (4.8)	94 (12.5)

Cough	41(14.1)	46 (19.4)	65 (28.5)	152 (20.2)

Abdominal pain/fullness	27 (9.3)	12(5.1)	29 (12.7)	68 (9.0)

Body weakness/malaise/pains	22 (7.6)	19 (8.0)	15 (6.6)	56 (7.4)

Vomiting	21 (7.2)	16 (6.8)	15 (6.6)	52 (6.9)

Flu/colds/running nose	17 (5.9)	7 (3.0)	22 (9.6)	46 (6.1)

Back pains/Joint pains/leg pains	17(5.9)	11(4.6)	15 (6.6)	43 (5.7)

Diarrhoea	9 (3.1)	18 (7.6)	20 (8.8)	47 (6.2)

Chest pain	7 (2.4)	10 (4.7)	15 (6.6)	32 (4.3)

Loss of appetite	6 (2.1)	4 (1.7)	9 (3.9)	19 (2.5)

Antenatal/medical check-up	0 (0.0)	11 (4.6)	1(0.4)	12 (1.6)

Most of the respondents (67.4%) reported to have their blood slide results within one hour. Of those, four (0.5%) patients received their laboratory results in less than 30 minutes, 282 (57.1% had their results after 30 minutes and 208 (42.1%) spent one hour to get their laboratory results. About 15.0% of the patients got results within two hours, 8.4% spent three hours and 7.8% took more than three hours to get their results. Half of the respondents (50.1%) felt that the waiting time was just right while 20.0%, 17.6% and 12.3% felt that the waiting time was long, too long and short, respectively. The largest proportion (90.0%) of the respondents was satisfied with laboratory services.

## Discussion

Laboratory malaria diagnosis is increasingly receiving much attention due to observed high rate of misdiagnosis [4] and adoption of more expensive anti-malarial drugs. However, previous studies have shown that the existing health laboratory system in Tanzania is incapable of maintaining good laboratory facilities that can support appropriate diagnosis of malaria and other infections that would lead to proper management of patients [6,21]. Although few facilities do provide laboratory services, insufficient trained laboratory personnel and clinicians' diagnostic practices are likely to be important obstacles in appropriate malaria diagnosis. These obstacles are common in other malaria endemic areas as reported from a study in Ghana [20].

This study has shown that the majority of laboratory personnel were not adequately trained and some were lacking professional qualifications. It was also shown that training opportunities and refresher courses for laboratory staff were rarely available while poor working environment was cited by most of the laboratory staff as another constraint for provision of better malaria diagnostic services. Most of the constraints mentioned by laboratory personnel involved in this study have also been reported elsewhere in sub-Saharan Africa [15,22].

In the current study, one third of the patients referred to the laboratory for malaria investigations were reported to have malaria. However, a survey to establish the accuracy of malaria microscopy in the same health facilities [6] revealed that the ability of the laboratory personnel in detecting malaria infection by microscopy was approximately 50% (Kappa value, κ = 0.489). This means that most of the patients reported to have malaria parasites did not actually have the parasites. The reported relatively high malaria slide positivity rate coupled with the observed presumptive diagnosis practices indicates high level of malaria misdiagnosis in the study health facilities.

Diagnosis of malaria using clinical presentation was evident in the current study. Nankabirwa and others [23] reported that such practices are highly sensitive in detecting malaria cases but their specificity was low. In the current study, less than half of the patients diagnosed to have malaria using clinical features were detected to have malaria parasites by microscopy. Likewise, the ability of fever to predict true cases of malaria was estimated at about one third of slide positive cases. Thus, managing fevers as malaria has a potential risk of malaria over-diagnosis and under-diagnosis of other febrile illnesses.

Despite the fact that laboratory malaria diagnostic services were available in all study health facilities, standard criteria for who to test was lacking and test results were underutilized in management of patients. Request for laboratory malaria test was unguided as some clinicians requested the test always while others ordered the test infrequently. Furthermore, less than half of the interviewed clinicians indicated to trust and use laboratory results whenever they were available while more than half indicated that test results have less influence on how they treat patients. Similar findings have been reported in Ghana [20]. This observation agrees with other studies which showed that with optimal laboratory testing clinicians' perceptions and practice remains one of the major barriers to effective laboratory use[14,16].

The majority of clinicians reported that results from their laboratories were reliable, but relatively few were always using them in malaria case management. It has been argued that, improvement in diagnostic sensitivity alone may not translate into improved patient care [24]. Thus, it is proposed that training of clinicians and appropriate supportive supervision may possibly change diagnostic behaviour of the clinicians [25]. In the current study, it was observed that some clinicians were not always requesting for malaria microscopy because laboratories service hours were relatively shorter than health care provision time. This might have provided a honest room for further justifying presumptive diagnosis. For the laboratories to support accurate disease diagnosis the working hours should as far as possible match with health care provision time.

Some studies [18,19] have shown that patients or their care-takers prefer laboratory investigations before anti-malarial drug prescription and patients with positive malaria test tend to be more satisfied. This was evident in the current study in which some patients demanded for malaria laboratory testing without requests from clinicians. Moreover, patients with positive malaria test were more satisfied compared with those with negative results. Thus, it can be argued that, the findings that some patient requested malaria test before anti-malarial drug prescription is an indication that the role of patients in malaria over-diagnosis is minimal. However, the decision by laboratory personnel to examine some patients and deny others who visited laboratory without prior consultation with clinicians was not clear. This is an indication of lack of standard operating procedures in the respective laboratory facilities. It was also evident that, the time spent by patients waiting for their laboratory results was not critical, as most of them were satisfied with laboratory services. This may be partly due to the fact that majority of the surveyed laboratories were using Field's stain [6], a technique that takes relatively shorter time than Giemsa technique and also since laboratory services were few, patients had to appreciate services they were getting from the few available laboratories. However, holding a patient for more than one hour waiting for laboratory results which will not guide appropriate drug prescription is unjustified.

This study was designed to collect information from clinicians and laboratory personnel working at the selected health facilities at the time of investigators' visit. The laboratory personnel and clinicians were aware that their diagnostic practices are being observed and this might have modified their actual practices. These factors affect generalization of the findings of this study. However, the fact that these findings corroborate those of other similar studies elsewhere may still have local or regional relevance. The findings highlight the complexity of the factors that might be responsible for poor quality and low utilization of laboratory results for malaria diagnosis.

## Conclusion

The findings show that laboratories in Tanzania have limited capacities for malaria diagnosis and the quality of malaria diagnostic services is more likely to be compromised by poorly trained, less motivated and over-worked laboratory personnel. Nearly three quarters of the clinicians were comfortable with the performance of their laboratories. It was evident that there were no standard criteria for requesting laboratory testing for malaria and laboratory results were not trusted by over half of the clinicians. Moreover, a large proportion of clinicians infrequently used test results for management of patients.

It was evident that patients preferred laboratory test before having anti-malarial drug prescription and those with positive malaria test were more satisfied with results. Although fever was found to be a preferred predictor of malaria, it was shown that treating all fevers as malaria resulted in over-diagnosis of malaria and masking investigation of other febrile illnesses. These findings suggest that since subjects to be tested for malaria were not well defined and test results were under-utilized, improvement of test results alone cannot necessarily lead to improved malaria management. Thus, updating malaria diagnostic guidelines, training of health providers and appropriate supportive supervision may possibly improve malaria diagnosis and utilization of test results. The current laboratory improvement programme aiming at improving the quality of malaria diagnosis in Tanzania should, therefore, take into consideration patients, clinicians and laboratory staff related factors, which most likely contribute to the performance of laboratories.

## Competing interests

The authors declare that they have no competing interests.

## Authors' contributions

RTR, DRSI, SMM, LEGM and JJM conceived and designed the study. DRSI, RTR, SMM and YAD were responsible for field data collections. DRSI, YAD and FT participated in analysis and interpretation of data. YAD drafted the manuscript. DRSI, LEGM, SMM, FT, YAD and JJM participated in revising the manuscript. All authors except RTR (deceased) read and approved the final manuscript.
